# Guided web app intervention for reducing symptoms of depression in postpartum women: Results of a feasibility randomized controlled trial

**DOI:** 10.1016/j.invent.2024.100744

**Published:** 2024-04-25

**Authors:** Pamela Franco, Marcia Olhaberry, Saskia Kelders, Antonia Muzard, Pim Cuijpers

**Affiliations:** a Doctoral Program in Psychotherapy, School of Psychology, Pontificia Universidad Católica de Chile, Av. Vicuña Mackenna 4860, Santiago, Chile; b Millennium Institute for Research in Depression and Personality (MIDAP), Santiago, Chile; c School of Psychology, Pontificia Universidad Católica de Chile, Av. Vicuña Mackenna 4860, Santiago, Chile; d Centre for eHealth & Wellbeing Research, Psychology, Health & Technology, Faculty of Behavioral, Management and Social Sciences, University of Twente, Drienerlolaan 5, 7522 NB Enschede, the Netherlands; e Optentia Research Unit, North-West University, VTC, South Africa; f School of Psychology, Finis Terrae University, Santiago, Chile; g Department of Clinical, Neuro and Developmental Psychology, Amsterdam Public Health Research Institute, Vrije Universiteit Amsterdam, De Boelelaan 1105, 1081 HV Amsterdam, the Netherlands; h Babeș-Bolyai University, International Institute for Psychotherapy, Cluj-Napoca, Romania

**Keywords:** Randomized controlled trial, Internet-based intervention, Guided self-help, Depression, Postpartum, Cognitive-behavioral therapy, Feasibility, Acceptability

## Abstract

**Background:**

Chile faces a significant postpartum depression prevalence and treatment gap, necessitating accessible interventions. While cognitive-behavioral internet-based interventions have proven effective in high-income countries, this field is underdeveloped in Chile. Based on the country's widespread use of digital technology, a guided 8-week cognitive-behavioral web app intervention named “*Mamá, te entiendo*” was developed.

**Objective:**

This study aimed to assess the acceptability and feasibility of “*Mamá, te entiendo*”, for reducing depressive symptomatology in postpartum women.

**Methods:**

Sixty-five postpartum women with minor or major depression were randomly assigned to either intervention or waitlist. Primary outcomes centered on study feasibility, intervention feasibility, and acceptability. Semi-structured interviews with a sub-sample enriched the understanding of participants' experiences. Secondary outcomes included mental health variables assessed at baseline, post-intervention, and 1-month follow-up.

**Results:**

Chilean women displayed great interest in the intervention. 44.8 % of participants completed the intervention. Participants reported high satisfaction and engagement levels, with interviewees highlighting the value of the intervention's content, exercises, and therapist's feedback. However, preliminary efficacy analysis didn't reveal a significant interaction between group and time for outcome measures.

**Discussion:**

This research represents a pioneering effort in Chile to evaluate an internet-based intervention for postpartum depression symptoms. The demonstrated feasibility and acceptability highlight the potential of integrating technology-driven approaches into mental health interventions. However, the intervention did not demonstrate superiority, as both groups exhibited similar positive progress in several outcomes. Therefore, the following research phase should involve a larger and more diverse sample to assess the intervention's effectiveness, identify influencing factors, and determine the individuals who benefit the most.

## Highlights

• Addresses Chile's significant treatment gap and high prevalence of postpartum depression
• Introduces Chile's first internet-based intervention targeting postpartum depression symptoms
• Demonstrates strong interest and satisfaction among Chilean postpartum women in the intervention
• Achieved a 44.8 % completion rate among participants in the intervention
• Both intervention and waitlist groups showed improvement in most mental health outcomes over time.

## 1 Background

Postpartum depression (PPD) poses a significant public health challenge, with a global prevalence of 16.7 % and a notably high rate of 38 % in Chile (Hahn-Holbrook et al., 2018). PPD is associated with impaired maternal functioning and compromised mother-infant bonding, which negatively affects the infant's development (Urizar and Muñoz, 2022). Despite the strategy of universal screening for depression symptoms implemented in the public health system in Chile for postpartum women (MINSAL, 2014), women's and healthcare providers' barriers hinder PPD recognition and treatment. Women face obstacles such as misconceptions about PPD and its treatments, stigma, fear of judgment, limited time and energy, and insufficient social support (Rojas et al., 2015; Irarrázaval, 2020). Meanwhile, public mental health services struggle with long waitlists, session frequency and duration that fall short of international standards, and high professional turnover (Rojas et al., 2015; De la Parra et al., 2019). Furthermore, only women diagnosed with PPD receive referrals for mental health care (MINSAL, 2013), leaving those who struggle with symptoms but don't meet the full diagnostic criteria without access to symptom relief and prevention of major depression.

The rise of e-mental health addresses the increasing demand for mental health support, utilizing internet-related tools for psychological internet-based interventions (IBIs). These interventions, delivered through websites or apps (Li et al., 2023), offer flexibility and cost-effectiveness (Baumann et al., 2020). Guided IBIs, those involving human support, have demonstrated superior effectiveness compared to unguided ones, especially for moderate to severe depression (Karyotaki et al., 2021). Despite promising outcomes for PPD-specific IBIs (Li et al., 2023), attrition rates remain a challenge in all IBIs (Linardon and Fuller-Tyszkiewicz, 2020), necessitating a focus on tailoring technology for usability, acceptability, and engagement (van Gemert-Pijnen et al., 2018).

Hence, an 8-week guided cognitive-behavioral IBI (“*Mamá, te entiendo*” [“*Mom, I get you*”]) was developed to alleviate depressive symptomatology in postpartum Chilean women, integrating aspects from mentalization theories. This development followed the CeHRes model, emphasizing a human-centered approach (van Gemert-Pijnen et al., 2018), and the randomized controlled trial aimed to assess the feasibility, acceptability, and preliminary outcomes of the newly developed intervention in Chilean women with minor or major depression. Additionally, qualitative exploration provided valuable insights into participants' perspectives, enriching the understanding of their experiences.

## 2 Method

The trial investigated in this study was registered in ClinicalTrials.gov (NCT05643898) in December 2022. Its procedure was approved by the Health Sciences Ethical Committee of Pontificia Universidad Católica (Santiago, Chile; protocol ID 210824004). A detailed protocol of its methods has been published (Franco et al., 2023).

### 2.1 Design

A mixed-methods feasibility and acceptability study with a two-arm, small-scale, randomized controlled trial was conducted. Postpartum women with minor or major depression were randomly assigned to the intervention (“*Mamá, te entiendo*”: MTE) or the control group (waitlist: WL). Primary outcomes included the study's feasibility, intervention's feasibility, and acceptability. Secondary outcomes encompassed depression, perceived social support, mother-infant bonding, and maternal self-efficacy. Data were collected at baseline (T0), post-intervention (T1: 8 weeks), and 1-month follow-up (T2: 12 weeks). Additionally, a qualitative evaluation with a subset of participants was conducted to gain insight into their experience with the intervention. Refer to the study protocol for more details on the study's design, procedures, instruments specifications, and definitions of variables and methods (Franco et al., 2023).

### 2.2 Participants

Participants were mothers of infants between 1 and 7 months with depressive symptomatology (defined by a score of ≥5 in the Patient Health Questionnaire-9 (PHQ-9)) and a minor or major depression diagnosis according to the Spanish version of the Mini-International Neuropsychiatric Interview (MINI). They had to be at least 18 years old, Chilean residents, Spanish-fluent, smartphone owners with internet access, and give informed consent. Participants were excluded if they reported illiteracy, a history of substance abuse, a history of bipolar or psychotic disorders, presented moderate or severe suicide risk (defined as a score > 1 on item 9 of PHQ-9 and a moderate or severe suicide risk level according to the MINI), and exhibited ongoing psychosis symptoms according to the MINI. Participants who were currently receiving psychological or psychopharmacological treatment were not excluded from the study to enhance ecological validity. In cases where major depression or anxiety disorder was identified, participants were encouraged to seek guidance from a healthcare professional or continue their mental healthcare. Participation in the trial did not involve monetary compensation, except for the acceptability interviews, for which participants were offered a 12 USD gift card.

#### 2.2.1 Recruitment, eligibility assessment, and randomization

Participants were recruited through social media ads that directed interested women to a website with the PHQ-9 questionnaire. A clinical psychologist then contacted potential participants to confirm their alignment with sociodemographic criteria and schedule an eligibility interview via video call. During the interview, sociodemographic and clinical history information was collected, and eligibility was assessed. After the interview, participants completed the T0 questionnaires.

Eligible participants were randomly allocated to MTE or WL, using Study Randomizer software (2017) with permuted block randomization and stratification for depression severity (minor or major depression) (block size of 6, 1:1 ratio). Researchers were masked to randomization and received participant allocation notification via email from an independent researcher. Participants and research personnel were unmasked post-randomization, except for T1 and T2 interviewers. Participants were emailed about their group assignment (MTE or WL). Additionally, the intervention group received instructions for web app registration.

### 2.3 Intervention condition: “Mamá, te entiendo” (“Mom, I get you”) (MTE)

Participants in the MTE group accessed an 8-week guided cognitive behavioral web app-based intervention addressing PPD symptomatology. The intervention incorporated elements from attachment and mentalization theories to enhance maternal sensitivity and foster a secure emotional connection between mothers and their babies (MacBeth et al., 2023). Its development was based on the CeHRes Roadmap (van Gemert-Pijnen et al., 2018) and employed a human-centered design with continuous evaluation cycles. Please refer to a separate paper for specific details about the intervention, its development and rationale (Franco et al., 2024).

MTE comprises six main and three optional sequential modules (see Table 2). Fictitious case examples featuring five mothers illustrate both depressive symptoms and therapeutic techniques. All modules except the two psychoeducational ones include homework. Personalized feedback from an e-coach (clinical psychologist) was provided within two working days. MTE also included infographics covering various postpartum topics, a shareable document offering insights on supporting loved one experiencing depression symptoms, a resources section, and a contact section for technical support. Participants were encouraged to add a shortcut to the web app on their smartphone home screen and progress at their own pace, receiving email reminders if inactive for seven days.

### 2.4 Waitlist condition (WL)

Participants in the WL underwent the same assessments as the intervention group, excluding those specific to the intervention. They were allowed to utilize standard mental health services during the waiting period. After the T2 assessment, they were granted access to the intervention.

### 2.5 Primary outcomes

The study's feasibility was evaluated across recruitment, eligibility, and study dropout rates, and required resources. Intervention feasibility was assessed through the Credibility and Expectancy Questionnaire (CEQ-6; 6 items) and utilization metrics. The Spanish-adapted CEQ-6 (Quero et al., 2017) includes items on treatment logic, expected satisfaction, recommendation, effectiveness, usefulness, and perceived aversiveness on a 0 to 10 scale. Descriptive analysis treated the first five items as a composite variable for the intervention's perceived credibility and expectancy of benefit, while item 6 indicated aversiveness. Utilization and adherence were gauged by participant registration, average module completion, percentages of completed main and extra modules, and exercise submission. Engagement in the intervention group was measured using the TWente Engagement with Ehealth Technologies Scale (TWEETS: Kelders et al., 2020; 9 items, range 0–4) at T1.

Intervention acceptability in the intervention group was assessed at T1 with the Spanish version of the Client Satisfaction Questionnaire (CSQ-8: Vázquez et al., 2019; 8 items, range 8–32), a self-report questionnaire measuring overall satisfaction. Following T2, five MTE participants, categorized as “high adherent” (completing all six main modules) and five “moderate adherent” (completing three to five modules), were randomly selected for semi-structured interviews to explore their experiences with the intervention.

### 2.6 Mental health outcomes

The Spanish version of the MINI (Ferrando et al., 2000) was used to establish diagnoses of mental health disorders. At T1 and T2, only the major depressive episode section was used, except when there was a positive response to question g, in which case, the suicide risk section was also utilized. The Spanish versions of the PHQ-9 (Diez-Quevedo et al., 2001; 9 items, range 0–27) and the Chilean version of the Edinburgh Postpartum Depression Scale (EPDS: Jadresic et al., 1995; 10 items, range 0–30) were utilized as self-report measures to evaluate depressive symptoms. The Spanish version of the Postpartum Bonding Questionnaire (PBQ: Garcia-Esteve et al., 2016; 25 items, range 0–125) assessed mother-infant bonding, while the Chilean Parental Assessment Scale (*Escala de Evaluación Parental*, EEP: Farkas-Klein, 2008; 10 items, range 0–10) measured maternal satisfaction and self-efficacy. The Chilean version of the Multidimensional Scale of Perceived Social Support (MSPSS: Oyarzun and Iriarte, 2020; 12 items, range 12–56) gauged perceived social support. Participants completed the PHQ-9 and EPDS at all assessment points, while the PBQ, EEP, and MSPSS were administered at T0 and T2. Data on mental healthcare service use were collected at all assessment points. More details on the instruments are available in the protocol paper (Franco et al., 2023).

### 2.7 Analyses

#### 2.7.1 Quantitative data

Descriptive statistics of recruitment and eligibility rates, participants' sociodemographic and clinical characteristics, data collection, study dropout, adherence to the intervention, intervention credibility and expectancy (CEQ-6), engagement with the intervention (TWEETS), and satisfaction with the intervention (CSQ-8), and for each secondary outcome at all assessment points, are reported. Adherence to the intervention was assessed by analyzing completion rates of modules and submitted exercises tracked within the intervention platform.

Baseline differences in demographic and clinical characteristics were examined using chi-square and *t*-tests. The preliminary efficacy of the MTE intervention was assessed based on the intention-to-treat (ITT) principle. Missing values, assumed to be missing at random, were imputed using the Groupwise multivariate imputation by chained equations (MICE) algorithm with fully conditional specification (van Buuren and Groothuis-Oudshoorn, 2011). The tutorial by Harrer et al. (2023) on evaluating randomized controlled trials in mental health research was used for data analysis.

The hypothesized superiority of MTE was tested for change in participants' depression symptoms (PHQ-9 and EPDS), mother-baby bonding, maternal self-efficacy, and perceived social support (PBQ, EEP, and MSPSS). Differences in change between study arms were assessed using univariate covariance analysis (ANCOVA) with baseline scores as covariates. T0 variables that showed significant differences between groups were also included as covariates. Effect sizes (Cohen's d) were calculated using the imputed dataset for between-group differences using the pooled MTE and WL groups' standard deviation (Leonhart, 2004). To calculate 95 % CIs, the formula by Rosnow and Rosenthal (2008) was used. According to Cohen (1988), d = 0.2 can be considered a small effect, d = 0.5 a medium effect, and d = 0.8 a large effect. A significance level of 0.05 (2-sided) was used for all analyses.

#### 2.7.2 Qualitative data

Semi-structured acceptability interviews were recorded and transcribed, excluding personal data, with recorded files deleted after transcription. Two independent coders (one project researcher and one external researcher) conducted thematic content analysis (Braun and Clarke, 2006) on the data, which involved comparing and condensing information addressing participants' perceptions of benefits, barriers, and facilitators. Finally, the coders carried out a discussion and findings triangulation.

## 3 Results

### 3.1 Participants characteristics

Table 1 presents participants' sociodemographic and baseline characteristics. The sample's average age was 32.53 (SD = 3.8; Range: 25–41). The majority were Chilean (93.8 %), first-time mothers (69.2 %), were in a cohabiting relationship (95.4 %), and possessed a university education (81.5 %). While 43.1 % were in current psychological therapy and 30.8 % in psychopharmacological treatment, 80 % had previously undergone psychological or psychopharmacological treatment.

**Table 1:** Participants' sociodemographic, clinical history, and baseline characteristics.

Baseline characteristics	Whole sample (*n* = 65)	MTE (*n* = 33)	WL (*n* = 32)	t/χ2	p
Age in years, M (SD)	32.53 (3.8)	33.21 (4.0)	31.84 (3.4)	1.46	0.14
Infant age in months, M (SD)	3.54 (1.8)	3.55 (1.7)	3.53 (2.0)	0.03	0.97
Parity, N (%)				0.98	0.32
First-time mother	45 (69.2)	21 (63.6)	24 (75.0)		
Has more children	20 (30.8)	12 (36.4)	8 (25.0)		
Planned pregnancy, N (%)				0.36	0.54
Yes	39 (60.0)	21 (63.6)	18 (56.3)		
No	26 (40.0)	12 (36.4)	14 (43.8)		
Education, N (%)				4.22	0.12
University education	53 (81.5)	29 (87.9)	24 (75.0)		
Vocational education	7 (10.8)	1 (3.0)	6 (18.8)		
High school	5 (7.7)	3 (9.1)	2 (6.3)		
Employment status before childbirth, N (%)				7.12	0.30
Employee	44 (67.7)	20 (60.6)	24 (75.0)		
Self-employed	9 (13.8)	7 (21.2)	2 (6.3)		
Student	6 (9.2)	3 (9.1)	3 (9.4)		
Unemployed	2 (3.1)	2 (6.1)	0		
Housewife	2 (3.1)	1 (3.0)	1 (3.1)		
Other	2 (3.1)	0	2 (6.2)		
Currently in psychological or psychopharmacological treatment	33 (50.8)	18 (54.5)	15 (45.5)	0.38	0.53
Currently in psychological treatment, N (%)	28 (43.1)	15 (45.5)	13 (46.4)	0.15	0.69
Currently in psychopharmacological treatment, N (%)	20 (30.8)	10 (30.3)	10 (31.3)	0.00	0.93
Prior psychological or psychopharmacological treatment, N (%)	52 (80.0)	31 (93.9)	21 (65.6)	8.14	0.00⁎
Depression diagnosis^a^, N (%)				0.50	0.47
Minor depression	8 (12.3)	5 (15.2)	3 (9.4)		
Major depression	57 (87.7)	28 (84.8)	29 (90.6)		
Dysthymia^a^, N (%)	6 (9.2)	4 (12.9)	2 (6.7)	0.66	0.41
Generalized anxiety disorder^a^, N (%)	40 (61.5)	18 (54.5)	22 (68.8)	1.38	0.23
Low-risk incidence of suicide^a^, N (%)	15 (23.1)	8 (24.2)	7 (21.9)	0.051	0.82
PHQ-9, M (SD)	13.01 (4.5)	11.66 (3.6)	14.40 (4.9)	−2.54	0.01⁎
EPDS, M (SD)	14.64 (4.1)	13.21 (3.7)	16.1 (4.0)	−3.02	0.00⁎
PBQ, M (SD)	26.50 (13.0)	25.75 (12.1)	27.28 (14.1)	−0.46	0.64
MSPSS, M (SD)	32.67 (9.0)	31.18 (7.0)	34.2 (10.6)	−1.34	0.18
EEP, M (SD)	5.99 (1.5)	6.24 (1.3)	5.7 (1.7)	1.29	0.19
CEQ-6, M (SD)	8.70 (1.5)	8.57 (1.7)	8.84 (1.2)	−0.69	0.48

Note. Abbreviations (alphabetical): CEQ-6 = Spanish Credibility and Expectancy Questionnaire-6 (first 5 items); EPDS = Edinburgh Postpartum Depression Scale; EEP = *Escala de Evaluación Parental*; M = Mean; MSPSS = Multidimensional Scale of Perceived Social Support; MTE = “*Mamá, te entiendo*” internet-based intervention; N = Number of participants; p: *p*-value; PBQ = Postpartum Bonding Scale; PHQ-9 = Patient Health Questionnaire 9-items; SD = Standard deviation; t: Statistic of independent *t*-test; W: waitlist group χ2 = Statistic of χ2-test.

For more detailed sociodemographic information, additional clinical history, and baseline characteristics, see Tables S1 and S2 in the Supplement.

⁎ *p* < .05.

**Table 2:** Modules of *“Mamá, te entiendo”*.

Module	Topic
0	Introduction to the intervention (without therapeutic content)
1	Psychoeducation on depression
2	How the cognitive-behavioral approach works
3	Identifying thinking errors
4	Cognitive restructuring techniques
5	Problem-solving
6	Behavioral activation
Guide for maintaining changes	Relapse prevention
Extra 1	Psychoeducation on anxiety
Extra 2	Exposure strategies
Extra 3	Communicational Skills

**Table 3:** Between-group effect sizes and results of analyses of covariance (ANCOVA) for the mental health variables.

			T0	T1				T2			
Variable	Analysis	Group	M (SD), n	M (SD), n	d (95 % CI)	F	p	M (SD), n	d (95 % CI)	F	p
PHQ9	ITT	MTE	11.67 (3.61), 33	9.79 (4.56), 33	−0.14 (−0.69 to 0.42)	1.7	0.63	9.97 (5.80), 33	−0.05 (−0.60 to 0.51)	0.97	0.87
		WL	14.41 (4.96), 32	11.61 (5.86), 32				11.69 (7.21), 32			
	CCA	MTE	11.67 (3.61), 33	9.45 (4.51), 22	−0.15 (−0.70 to 0.39)	2.9	0.57	9.50 (5.57), 22	−0.09 (−0.63 to 0.46)	2.27	0.75
		WL	14.41 (4.96), 32	11.83 (5.84), 30				12.07 (7.18), 30			
EPDS	ITT	MTE	13.21 (3.74), 33	11.18 (4.47), 33	0.03 (−0.45 to 0.51)	2.63	0.89	10.06 (4.84), 33	−0.08 (−0.61 to 0.45)	2.19	0.77
		WL	16.12 (4.03), 32	13.18 (6.08), 32				12.12 (6.19), 32			
	CCA	MTE	13.21 (3.74), 33	10.86 (4.53), 22	−0.05 (−0.55 to 0.44)	4.73	0.82	9.50 (4.70), 22	−0.20 (−0.75 to 0.35)	4.01	0.46
		WL	16.12 (4.03), 32	13.60 (5.96), 30				12.40 (6.16), 30			
EEP	ITT	MTE	6.25 (1.39), 33					6.87 (1.35), 33	0.08 (−0.36 to 0.53)	1.23	0.72
		WL	5.74 (1.75), 32					6.47 (1.77), 32			
	CCA	MTE	6.25 (1.39), 33					6.79 (1.27), 22	0.21 (−0.27 to 0.69)	1.44	0.39
		WL	5.74 (1.75), 32					6.34 (1.75), 30			
PBQ	ITT	MTE	25.76 (12.14), 33					19.98 (8.84), 33	−0.03 (−0.48 to 0.42)	0.23	0.89
		WL	27.28 (14.11), 32					20.94 (12.34), 32			
	CCA	MTE	25.76 (12.14), 33					20.68 (8.74), 22	−0.09 (−0.57 to 0.40)	0.06	0.72
		WL	27.28 (14.11), 32					21.37 (12.49), 30			
MSPSS	ITT	MTE	31.18 (7.10), 33					36.33 (6.37), 33	0.07 (−0.39 to 0.54)	0.28	0.76
		WL	34.22 (10.66), 32					37.27 (10.16), 32			
	CCA	MTE	31.18 (7.10), 33					36.09 (6.18), 22	0.07 (−0.40 to 0.54)	0.22	0.76
		WL	34.22 (10.66), 32					37.03 (10.25), 30			

Note. Abbreviations (alphabetical): CCA = Complete Cases Analyses; d = Cohen's d; EPDS = Edinburgh Postpartum Depression Scale; EEP = Escala de Evaluación Parental; ITT = Intent-to-treat Analyses; M = mean score; MSPSS = Multidimensional Scale of Perceived Social Support; MTE = “*Mamá, te entiendo*” Internet-based intervention; p = p-value; PBQ = Postpartum Bonding Scale; PHQ-9 = Patient Health Questionnaire 9-items; SD = Standard deviation; WL = waitlist; F = F-statistic.

Among participants, 87.7 % had major depression, and 12.3 % had minor depression, according to the MINI. There weren't significant differences between the conditions in terms of sociodemographic and clinical variables at T0, except for higher mean scores in the PHQ-9 (MTE: M = 11.66, SD = 3.6; WL: M = 14.40, SD = 4.9; *t* = −2.54, *p* = .01) and EPDS (MTE: M = 13.21, SD = 3.7; WL: M = 16.1, SD = 4.0; *t* = −3.02, *p* = .00) in the WL. Additionally, more participants from MTE reported prior mental health treatment, whether psychological or psychopharmacological treatment (MTE: 31/33 & WL: 21/32; Cramer's V = 0.35, *p* = .004).

### 3.2 Study feasibility

#### 3.2.1 Recruitment and eligibility

**Fig. 1:**
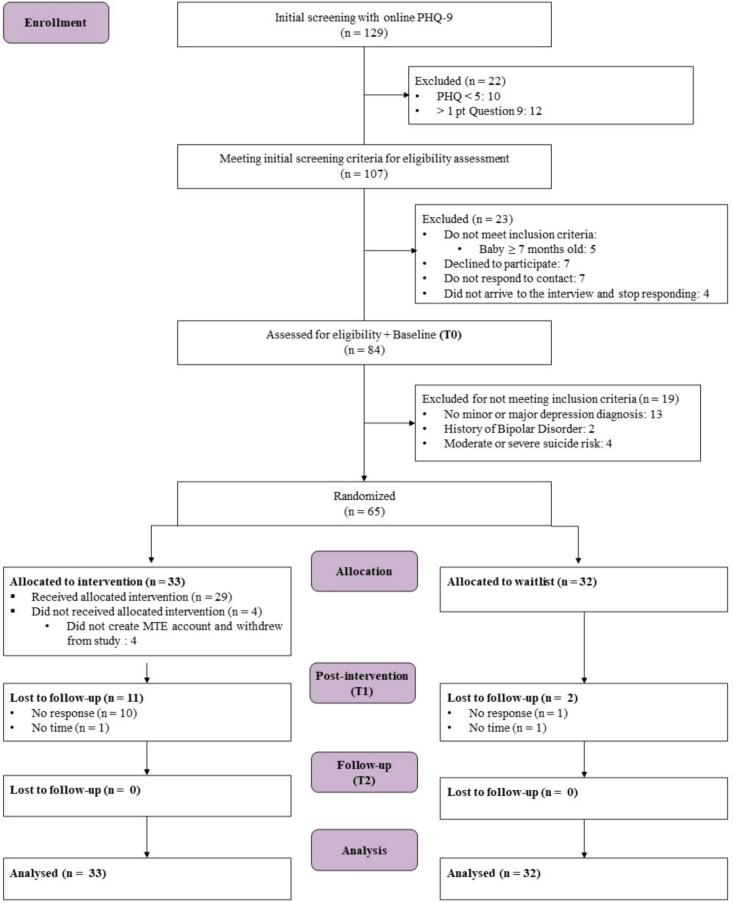
Participant flow through study.

Recruitment for the study began in February 2023. The last follow-up was completed on June 22, 2023. Acceptability interviews were conducted between June 20 and July 17, 2023. Fig. 1 depicts the participant flow and attrition throughout the trial as outlined in the CONSORT diagram. The recruitment initiative resulted in 129 postpartum women engaging in the initial screening by completing a questionnaire within ten days. After eligibility interviews, 65 participants were randomized, with 33 allocated to MTE intervention and 32 to WL.

#### 3.2.2 Study dropout

The study had a dropout rate of 20.0 % at T1, with no further dropouts at T2. Dropout rates were unbalanced between MTE (11) and WL (2). Participants who dropped out scored significantly higher in terms of maternal satisfaction and self-efficacy, as measured by the EEP (M = 6.73, SD = 0.7), in comparison to participants who completed the assessments (M = 5.80, SD = 1.6), t(63) = −2.90, *p* = .006. Additionally, participants who dropped out scored significantly lower in mother-baby bonding problems, as measured by the PBS (M = 18.30, SD = 6.8), compared to participants who completed the assessments (M = 28.55, SD = 6.8), t(63) = −2.64, *p* = .000, with a larger proportion falling within the no bonding disorder range (Cramer's V = 0.33, *p* = .007). Moreover, primiparas were more likely to complete the assessment than mothers with multiple children (Cramer's V = 0.30, *p* = .045).

#### 3.2.3 Resources needed to complete the study

Potential participants took about 5 min to complete the initial assessment. T0 lasted approximately 45 min, while the T1 and T2 assessments took 15–20 min each. Two clinical psychologists conducted the interviews. Completing data collection for all participants within each assessment period took around four weeks, considering rescheduling by participants. *E*-coaches spent between 5- and 15 min providing feedback for each exercise. Participants submitted 180 exercises, equivalent to approximately 30 h of e-coaching (about 1 h per participant, taking into account all registered participants who used the web app).

### 3.3 Intervention feasibility

#### 3.3.1 Intervention's credibility and expectancy of benefit

The CEQ-6 (Items 1–5) yielded an average score of 8.70 (SD = 1.5). A less favorable perception of mother-baby bonding was found to be associated with a reduced tendency to view the intervention as believable/logical and beneficial/helpful (*r* = 0.42, *p* = .00). No significant associations were observed with other variables. Participants did not perceive the intervention as aversive; the score for this question had a mean of 0.60 (SD = 1.73) on a scale ranging from 0 to 10.

#### 3.3.2 Intervention adherence

**Fig. 2:**
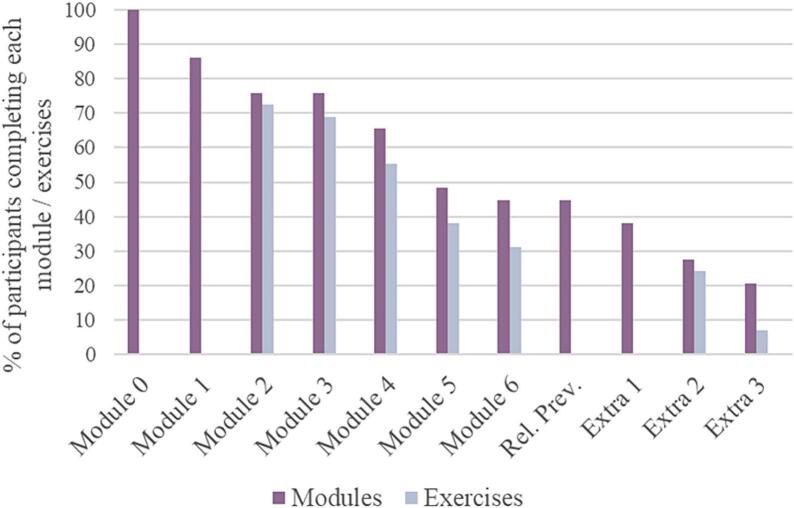
Percentage of participants completing each module and the module's exercises (*n* = 29). Note. This figure depicts the participant completion percentages for each sequential module (purple bars), including the introductory module 0 and the relapse prevention guide, and the rates of participant exercise submissions (grey bars).

Among the MTE group participants, four never registered on the web app and consequently withdrew from the study. Of the 29 participants who registered for the web app, 13 (44.8 %) completed the six core modules, and 6 (20.6 %) finished all available modules. On average, participants completed approximately 3.97 core modules (SD = 2.2) and 0.83 extra modules (SD = 1.2). Concerning the exercises, 25 participants (86.2 %) submitted at least one exercise, with 9 participants (31.0 %) submitting exercises for all core modules. Out of the 13 participants who completed all six core modules, nine (69.2 %) submitted exercises for each of those modules. Refer to Fig. 2 for participants' completion rates.

No significant differences were found in T0 measures between participants who completed all six main modules and those who didn't (including, in this group, the ones who didn't register into the web app). However, higher scores on both PHQ-9 and EPDS were associated with less modules completed (*r* = −0.48, *n* = 29, p = .00 and *r* = −0.40, n = 29, *p* = .2, respectively). Nonetheless, higher scores in the PBQ were associated with a higher exercise submission (*r* = 0.41, n = 29, *p* = .02).

All participants from the MTE group who dropped out of the study had completed between zero and three modules, with only one of them submitting an exercise.

#### 3.3.3 Engagement with the intervention

The mean total TWEETS score was 3.05 (SD = 0.58), with only one participant assigning the lowest possible score to one item. The sample exhibited a left-skewed distribution (skewness = −0.96, SE = 0.49). The items, ranked from highest to lowest mean, are as follows: “MTE helped me to get more insight into my thoughts and emotions” (3.68), “I enjoyed seeing the progress I made in MTE” (3.36), “MTE motivated me to improve my mood” (3.27), “MTE fits me as a person” (3.23), “I enjoyed using MTE” (3.14), “MTE made it easier for me to work on improving my mood” (3.09), “MTE takes me little effort to use” (3.00), “I was able to use MTE as often as needed to improve my mood” (2.77), and “MTE was part of my daily routine” (2.00). See Table S3 in Supplements for TWEETS item response frequency. Higher scores in the TWEETS were associated with more submitted exercises (*r* = 0.49, p = .02). However, there was no association between the number of modules completed and the TWEETS.

### 3.4 Intervention acceptability

#### 3.4.1 Satisfaction with the intervention

The total CSQ-8 score mean was 26.68 (SD = 4.3), with no participant giving the lowest possible score for any item. The sample showed a distribution that skewed to the left (skewness = −0.79, SE = 0.49). The items, ranked from highest to lowest mean (in a range from 1 to 4), were as follows: Overall satisfaction (3.59), Recommend to a friend (3.59), Amount of help (3.50), Come back (3.45), Quality of service (3.36), Deal with problems (3.32), Type of service (3.18), Solved problems (2.68). See Table S4 in Supplements for CSQ-8 item response frequency. Higher scores in the CSQ-8 were associated with more exercise submission (*r* = 0.50, *p* = .01), but there was no association between the number of modules completed and the CSQ-8.

### 3.5 Qualitative intervention feasibility and acceptability outcomes

Lastly, five participants from the “high adherence” group (E1, E2, E3, E5, E6) and one from the “moderate adherence” group (E4) participated in acceptability interviews (codes to be referenced in subsequent results). The remaining four participants who were invited cited time constraints as the reason for not participating. Thematic analysis generated four key themes (content, exercises & e-coach's feedback, user experience and delivery format, and helpfulness) and 15 subthemes (highlighted in bold within the text). Table S5 in Supplements provides a summary of key subthemes, highlighting facilitators and barriers to the use of MTE.

#### 3.5.1 Theme 1: MTE content

The **content** was perceived as “*comprehensive*” (E1, E2, E3), with “*nothing superfluous in it*” (E2, E5, E6), “*down-to-earth*” (E1, E2), “*useful*” (E4, E5, E6), and “*engaging*” (“*I was eager to progress to find out what the next module was going to say*”, E6). The language was perceived as “*clear*” (E1, E5), “*supportive*” (E1), and “*approachable*” (*“Like reading a psychologist or a friend talking to you*”, E1; E3). Also, the techniques taught are seen as helpful and easy to use (E1, E2).

Two participants (E1, E3) mentioned sometimes the content felt lengthy or more complex when advancing in the modules, requiring greater concentration and time dedication for comprehension and analysis. These difficulties become even more relevant due to the context in which the mothers find themselves, as all of them mentioned accessing the intervention at night after the baby fell asleep. Therefore, they were significantly tired at the time of accessing MTE, and this adds to the perception of having reduced attention, with two mothers mentioning they were having “*baby brain*” (“… *the baby brain, you know, sometimes I had to read it twice*”, E1; E3).

All the interviewees highly appreciated the inclusion of **fictional moms** exemplifying the content. The examples were perceived as “*real*” (E1), and participants felt “*represented*”/“*identified*” with their stories (E1, E2, E3, E4, E5, E6). It made the content “*more illustrative*” (E1) and “*easier to understand*” (E3). Participants valued that it helped to “*normalize experiences*” they have or could live (E3, E4, E6) and to feel less isolated in their experiences. It was appreciated that various forms of family compositions and situations were displayed.

#### 3.5.2 Theme 2: MTE exercises and e-coach's feedback

All interviewees appreciated the inclusion of **exercises**. They mention that they are “*useful*” (E5) and a good complement to the information/content to put it into practice. Furthermore, the exercises “*help internalize the content*” (E4), “*assist in reflection*” (E1), and “*aid in reading the content more attentively*” (E6).

Likewise, all the interviewees valued the **e-coach's feedback**. The e-coach was perceived as “*approachable*” (E1, E5, E6), “*empathetic*” (E1, E3, E4, E5), and “*kind*” (E4, E6). The feedback provided is seen as “*helpful*” (E2) and “*clear*” (E1, E2, E3), “*offering guidance without imposing ideas*” (E1, E6). It made the intervention “*personalized*” (E1, E3, E6) and made mothers felt accompanied (E2, E3, E5, E6). The mothers felt that someone cared about them (E2, E5, E6) and would understand them without judgment (E5, E6, E1). Participants mentioned that the feedback motivated them to continue using MTE and submitting exercises (“*When I realized there was a response and that it was guided by what you had answered, um, you feel more accompanied, you feel like reading more or doing more exercises*”, E3), making them feel like they were “*doing it right*” (E4, E6), and feeling “*validated*” (E6).

#### 3.5.3 Theme 3: user experience and delivery format of MTE

Overall, the interviewees expressed positive perceptions regarding their **user experience** with MTE. They perceived the intervention as a “*support*” (E1, E2, E5, E6), as “*accompanying*” (E3), and as “*an app for you*” (E1, E4, E5). It also felt approachable, and this is seen as a surprise because of the format (“*It was super positive, I was surprised..., how an app can be so approachable; because it is like a format, one can think that it is a bit impersonal*”, E1). Some mothers also mentioned perceiving the moment they access MTE as a “*moment of self-care*” (E1, E2, E5).

Regarding the **design** and aesthetics, the interviewees described the interface as “*pretty*”, “*subtle*”, “*visually pleasing*,” and “*approachable*” (E2, E3, E6). All of them describe MTE as “*intuitive*” and “***user-friendly***” (“*It was easy for me to use and navigate it, to go look for the modules and things, I didn't get tangled up at all*”, E1). Additionally, all appreciated the inclusion of illustrations and photos, which they perceived as “*pretty*” (E6), “*pleasant*” (E1), relevant and complementary to the written information (E4), and “*making the reading more interesting*” (E3).

The interviewees made some suggestions for improvements to the web app design. The most common request was improving progress tracking within the modules (E3, E4, E5), as the web app shows you which is the last module you accessed but not inside the module you left. Additionally, some participants suggested enhancing the design by reducing text density by including more visual aids (E1, E2).

When giving their opinion about MTE being a **web app** instead of a mobile app, most participants did not observe significant differences that would hinder access because they used the shortcut as if it were a mobile app icon. All the participants who completed T1 assessments, except for two, reported creating a shortcut for the web app on their smartphone screen. Regarding the **duration of the intervention**, four interviewees mentioned that the period was adequate (E1, E3, E5, E6). One mother said she finished it before schedule and had time to reread some modules (E6). Two mothers mentioned that they needed more time (E2, E4). As for the **frequency of accessing** the web app, it varies from every other day (E2), once a week (E4, E5), to once or twice a week (E6). Two mothers mentioned the app “*became part of the routine*”, and they tried to organize their schedule to be consistent (E1, E6).

#### 3.5.4 Theme 4: MTE helpfulness

Regarding MTE's objectives, all the interviewed mothers acknowledge that the intervention contributed to their mental health (“*It was a good experience for me…it helped me a lot, and I hope it helps many mothers*”, E3); “*From everything, I took something I learned, and it helped make my* [postpartum] *process a more bearable*”, E2).

A relevant aspect mentioned by the interviewees contributing to their mental health is the role MTE played in the process of **reflective understanding** in situations associated with their motherhood experience, emerging emotions, and depression and anxiety symptoms (“*It helped me be more understandable with myself and my baby*”, E3, “*It helped me understand some things that were happening to me or that I was feeling*”, E5; E6). One mother mentioned the app helped her to recognize she was having anxiety symptoms and look for professional help (E2).

The interviewees also mentioned MTE helped them understand their negative feelings or thoughts towards motherhood within the context of the situation where they emerged. This **normalization** process enabled them to **validate their feelings or thoughts** (E6, E1, E3, E4) and “***alleviate the guilt***” (E6, E5, E4) that came with the appearance of those feelings or thoughts (i.e. when thinking of missing the life they had before having their baby when feeling overwhelmed). Identification with fictional mothers also contributed to normalization of experiences and feeling less isolated and inadequate (E4; “*Knowing that it happens to everyone reduces guilt; you feel less alone, more accompanied*”, E5). Moreover, one participant mentioned that this process helped her regulate her emotions “*and not let them cloud your judgment*” (E1).

On the other hand, the mothers perceive the intervention contributed to the **modification of dysfunctional thoughts**, mainly related to the demands of the postpartum period, caregiving, and parenting. The interviewees mentioned that the intervention helped them “*identify their thinking errors and challenge them*” (E1), “*put things into perspective or context*” (E2), “*let go of expectations*” (E1), “*reduce self-imposed pressure, and care less about what others think*” (E6). One highlighted the technical usefulness of reducing goals to avoid the frustration of not meeting expectations (E2), and two mothers emphasized the “best friend technique” for treating themselves kindlier (E2, E3). One mother highlighted incorporating a relapse prevention section to be attentive to red flags (E5).

### 3.6 Mental health outcomes

At T1 and T2, there were no significant differences in the prevalence rates of minor depression, major depression, and no depression diagnoses between MTE and WL. Specifically, at T2 in MTE, 5 participants (22.7 %) received a major depression diagnosis, 2 (9.1 %) were diagnosed with minor depression, and 15 (68.1 %) did not meet the depression diagnosis criteria. In contrast, at T2 in WL, 10 participants (33.3 %) were diagnosed with major depression, 1 (3.3 %) with minor depression, and 19 (63.3 %) did not meet the depression diagnosis criteria. See Table S6 in. Supplements for a detailed breakdown of MINI depression diagnoses at all assessments by group.

At T1, no significant differences were observed between MTE and WL in depressive symptoms (PHQ-9: F = −1.70, *p* > .05, d = −0.14, 95 % CI [−0.69, 0.42]; EPDS: F = 2.63, p > .05, d = 0.03, 95 % CI [−0.45, 0.51]). This pattern persisted at T2 (PHQ-9: F = 0.9, p > .05, d = −0.05, 95 % CI [−0.60, 0.51]; EPDS: F = 2.19, p > .05, d = −0.08, 95 % CI [−0.61, 0.45]). Additionally, no significant difference was found between groups for EEP (F = 1.23, p > .05, d = 0.08, 95 % CI [−0.36, 0.53]), PBQ (F = 0.23, p > .05, d = −0.03, 95 % CI [−0.48, 0.42]), and MSPSS (F = 0.28, p > .05, d = 0.07, 95 % CI [−0.39, 0.54]) at T2. Between-group effect sizes and the results of ITT and completer case analyses are detailed in Table 3. Both groups significantly improved in EPDS, EEP, and PBQ throughout the study. Table S6 in the Supplement presents paired *t*-test results and effect sizes for intragroup changes from T0 at T1 and T2.

#### 3.6.1 Mental healthcare services use

The proportion of participants currently undergoing mental health treatment at T1 and T2 showed no significant differences between the groups. However, the WL exhibited a significantly higher proportion of women initiating mental health treatment from T0 to T1 (MTE: 4, WL: 11; Cramer's V = 0.38, *p* = .009).

## 4 Discussion

This study assessed the feasibility and acceptability of “*Mamá, te entiendo*”, a guided IBI for PPD symptoms, using a pilot randomized controlled trial. Within ten days, 129 women completed the initial screening, indicating a substantial interest among Chilean women in a web app format for enhancing postpartum mental health. Notably, this delivery format is novel in the country; participants lacked prior experience with similar interventions, and most had experience with traditional approaches. The intervention scored high on credibility and expectancy of benefit, with no discernible differences based on prior or current experience with conventional mental health treatment.

Concerning adherence, 39.3 % of participants in the intervention group and 44.8 % of those who registered into MTE completed all six central modules, closely resembling the rates reported. in perinatal depression IBIs with comparable length and therapeutic support. Forsell et al. (2017), Heller et al. (2020), and Suchan et al. (2022) reported completion rates ranging from 46 % to 50 % with a similar number of modules. Other studies on PPD IBIs have reported higher adherence rates; however, these studies differ from our's in their design, such as excluding participants receiving other mental health treatment, or in the intervention intensity, such as including e-coach phone calls or offering fewer modules (Danaher et al., 2013; Milgrom et al., 2016, Milgrom et al., 2021; Pugh et al., 2016; Loughnan et al., 2019). Regarding guided IBIs for depression in general, Moshe et al. (2021) found a 56.36 % completion rate in a meta-analytic review that included 49 studies. Despite our efforts to design the intervention using a human-centered approach and providing coaching support, achieving high adherence remains challenging. This is particularly significant as research indicates a substantial connection between adherence and outcomes (Moshe et al., 2021). However, the completion of the intervention by around 40 % of women experiencing PPD symptoms, most of them with major depression diagnosis, represents a meaningful step in improving access to cost-effective interventions for preventing or treating this disorder.

To enhance the intervention's user experience, adherence, and engagement for the definitive trial, we plan to incorporate features such as push notification reminders (Bidargaddi et al., 2018), an autosave function for exercises, reduced text density, improved progress tracking within the modules, and a night mode. Given the observed high comorbidity between depression symptoms and generalized anxiety disorder, we will modify the access to “extra” anxiety-related modules, allowing women to access them at any stage of the intervention, irrespective of their progress in the core modules. Personalizing the experience, including a mood monitoring feature (Torous et al., 2020) and sending an email from the corresponding e-coach to introduce herself and suggest modules based on the psychological evaluation results, could further improve user engagement.

The correlation between study dropout and heightened maternal self-efficacy and a positive bond with the baby, but not with depression severity, suggests an interesting pattern. One interpretation is that mothers feeling more competent and connected with their babies may be less overwhelmed by depressive symptoms, potentially leading to lower motivation for an intervention targeting these symptoms. Conversely, greater depression severity was associated with a lower completion rate of intervention modules, suggesting that severe symptoms might hinder engagement. Notably, a less favorable perception of the bond with the baby was linked to more submitted exercises, suggesting that mothers facing challenges in their relationship with their baby may be more motivated to improve this situation.

Satisfaction with the intervention and engagement were positively correlated with the submission of more exercises, facilitating increased interaction with the e-coach. As exercises allowed mothers to address personally relevant issues, they likely experienced a more personalized intervention, motivating them to engage and establish a therapeutic relationship with the e-coach. During interviews, mothers emphasized the pivotal role of the triad—consisting of content, exercises, and e-coach feedback—as key, complementary, and indispensable components of the intervention. Interviewees highly valued the intervention's perception as close support, especially during a postpartum period characterized by solitude, emotional ambivalence, and uncertainties about their parenting efforts. In fact, reviews of guidance's role in IBIs suggest that adding guidance may enhance treatment utilization and adherence while increasing overall treatment satisfaction (Baumeister et al., 2014; Shim et al., 2017).

The study primarily focuses on feasibility and acceptability, and although a preliminary efficacy analysis was conducted, no significant differences emerged between the MTE and WL groups across assessed variables. One potential explanation for this study's observed lack of effect could be the substantial improvement noted in the control group, suggesting that the positive changes in both groups might be attributed to spontaneous recovery or other effects related to study participation. In fact, evidence shows that the reduction of depressive symptoms over time among control conditions in research trials is not uncommon (Schienle and Jurinec, 2023; Tong et al., 2023). In the study by Suchan et al. (2022) on an IBI for PPD, a superiority effect of the intervention was observed, yet significant improvement was also noted in the treatment-as-usual group. Another potential explanation for our study's findings is the participants' utilization of additional mental health services. Approximately half of the participants in both groups were already receiving mental health treatment, and a notably higher proportion of participants in the waitlist group initiated mental health treatment from baseline to post-intervention.

Furthermore, since guided IBIs offer a cost-effective alternative that requires significantly less time than traditional individual therapies (Baumann et al., 2020), it would be beneficial to assess how the intervention could be integrated into a stepwise treatment model. MTE might serve as an essential resource for women with minor or subthreshold depression, providing early intervention, a supplement to standard treatment, or support during wait times for healthcare services, thereby enhancing the existing mental health framework. Future research focused on identifying which individuals gain the most from the intervention will aid in determining how it can optimally complement existing healthcare services.

### 4.1 Limitations

Several limitations of this study should be considered. First, the recruitment strategy attracted participants predominantly from middle to high-income backgrounds with higher education and stable partner relationships, potentially limiting the generalizability of the findings to the broader Chilean population. Future studies could benefit from diversifying recruitment through clinical settings to include participants from various health centers, particularly those with lower economic resources. Secondly, a notable dropout rate in the intervention group, particularly early in the intervention stage, suggests potential barriers and emphasizes the need for follow-up assessments to explore participant's experiences. Hence, caution is advised when interpreting satisfaction and engagement results. Thirdly, interviews were conducted with a small subsample, most of whom had completed the intervention, which may limit the generalizability of qualitative findings. Nonetheless, it's noteworthy that the comments from the moderate adherent participant did not differ from those of the high adherent participants. Fourth, we did not include low-adherence participants in the interviews, which is a limitation as they might have provided insights into barriers to engagement. This exclusion was due to this group's high study dropout rate and the practical challenges of re-engaging them long after their last app interaction. Future research should aim to include perspectives from participants with varying adherence levels to understand the engagement factors in the intervention fully. Fifth, with a small sample size, the study's nature as a feasibility study restricts the ability to draw conclusions about intervention effectiveness. Sixth, we did not collect information on the details of the mental health treatment that participants were undergoing, such as psychotropic medications, dose changes, or the frequency of psychotherapy sessions. Finally, the brief follow-up period limits the assessment of intervention effects beyond 12 weeks post-enrollment.

### 4.2 Conclusions and future remarks

This study represents a pioneering effort to evaluate an IBI aimed at alleviating depressive symptoms in postpartum women in Chile, marking a first in Latin America. The brief recruitment period underscored a substantial interest among Chilean women in technology-mediated solutions for postpartum mental health. Despite notable attrition rates, the alignment of these percentages with those from similar studies in higher-income countries is seen as a positive indicator. Participants expressed high satisfaction and engagement with the intervention, appreciating its content and guidance. They also reported beneficial impacts on their mental health and motherhood experiences. Future research will focus on assessing the intervention's effectiveness in a larger and more diverse sample, exploring factors that may influence its efficacy, and identifying specific groups that could benefit most.

## Declaration of competing interest

The authors declare that the study was conducted in the absence of any commercial or financial relationships that could be construed as a potential conflict of interest.

## Appendix A Supplementary data

Supplementary material Image 1
