# Supplementary material for: Guided web app intervention for reducing symptoms of depression in postpartum women: Results of a feasibility randomized controlled trial
Source: Internet Interv. 2024 Apr 25;36:100744. doi: 10.1016/j.invent.2024.100744 (PMC11067323; doi:10.1016/j.invent.2024.100744)
Supplement: Supplementary file 1 — Supplementary material [file mmc1.docx]

**SUPPLEMENT**

*Web App Intervention for Reducing Depressive Symptoms in Postpartum Women: A Feasibility Randomized Controlled Trial*

**Table S1**

*Participants’ socio-demographic characteristics.*

| Variable | Whole sample  (n= 65) | MTE  (n= 33) | Waitlist  (n= 32) |  | p |
| --- | --- | --- | --- | --- | --- |
|  | M (SD) | M (SD) | M (SD) | t |  |
| Age (years)  Range | 32.53 (3.8)  25-41 | 33.21 (4.0) | 31.84 (3.4) | 1.46 | .14 |
| Infant age (months)  Range | 3.54 (1.8)  1-6 | 3.55 (1.7) | 3.53 (2.0) | .03 | .97 |
| Weeks of gestation  Range | 38.20 (1.3)  34-41 | 38.06 (1.4) | 38.34 (1.0) | -.87 | .38 |
|  |  |  |  |  |  |
|  | N (%) | n (%) | n (%) | χ2 |  |
| Parity |  |  |  | .98 | .32 |
| First-time mother | 45 (69.2) | 21 (63.6) | 24 (75.0) |  |  |
| Has more children | 20 (30.8) | 12 (36.4) | 8 (25.0) |  |  |
| Planned pregnancy |  |  |  | .36 | .54 |
| Yes | 39 (60.0) | 21 (63.6) | 18 (56.3) |  |  |
| No | 26 (40.0) | 12 (36.4) | 14 (43.8) |  |  |
| Had a pregnancy complication | 22 (33.8) | 8 (24.2) | 14 (43.8) | 2.76 | .09 |
| The mother had a health complication during labor or postpartum |  |  |  | 3.62 | .30 |
| During labor | 20 (30.8) | 10 (30.3) | 10 (31.3) |  |  |
| Postpartum | 4 (6.2) | 1 (3.0) | 3 (9.4) |  |  |
| During labor & postpartum | 2 (3.1) | 0 | 2 (6.3) |  |  |
| The baby had a health complication during labor or after birth |  |  |  | .51 | .91 |
| During labor | 22 (33.8) | 11 (33.3) | 11 (34.4) |  |  |
| After birth | 8 (12.3) | 5 (15.2) | 3 (9.4) |  |  |
| During labor & after birth | 2 (3.1) | 1 (3.0) | 1 (3.1) |  |  |
| Has a chronic disease | 18 (27.7) | 10 (30.3) | 8 (25.0) | .22 | .63 |
| Relationship status |  |  |  | 3.05 | .38 |
| In a relationship, living together | 62 (95.4) | 32 (97.0) | 30 (93.8) |  |  |
| In a relationship, not living together | 1 (1.5) | 0 | 1 (3.1) |  |  |
| Single | 1 (1.5) | 0 | 1 (3.1) |  |  |
| Other (In separation process, living together) | 1 (1.5) | 1 (3.0) | 0 |  |  |
| Education |  |  |  | 4.22 | .12 |
| University education | 53 (81.5) | 29 (87.9) | 24 (75.0) |  |  |
| Vocational education | 7 (10.8) | 1 (3.0) | 6 (18.8) |  |  |
| High School | 5 (7.7) | 3 (9.1) | 2 (6.3) |  |  |
| Employment status before childbirth |  |  |  | 7.12 | .30 |
| Employee | 44 (67.7) | 20 (60.6) | 24 (75.0) |  |  |
| Self-employed | 9 (13.8) | 7 (21.2) | 2 (6.3) |  |  |
| Student | 6 (9.2) | 3 (9.1) | 3 (9.4) |  |  |
| Unemployed | 2 (3.1) | 2 (6.1) | 0 |  |  |
| Housewife | 2 (3.1) | 1 (3.0) | 1 (3.1) |  |  |
| Other (Student & Self-employed / Student & Employee) | 2 (3.1) | 0 | 2 (6.2) |  |  |
| Household income |  |  |  | 6.53 | .25 |
| $320.000 - $499.999 | 1 (1.5) | 1 (3.0) | 0 |  |  |
| $500.000 - $750.000 | 3 (4.6) | 2 (6.1) | 1 (3.1) |  |  |
| $750.001 - $ 1.125.999 | 6 (9.2) | 1 (3.0) | 5 (15.6) |  |  |
| $1.126.000 - $ 3.000.000 | 32 (49.2) | 18 (56.3) | 14 (43.8) |  |  |
| $3.000.000 | 21 (32.3) | 11 (52.4) | 10 (47.6) |  |  |
| Did not answer | 2 (3.2) | 0 | 2 (6.3) |  |  |

*Note.* Abbreviations (alphabetical): χ2 = Statistic of χ2-test; M = Mean; n = Number of participants; p = p-value; SD = Standard deviation; t = Statistic of independent t-test.

**Table S2**

*Participants’ clinical history variables and baseline characteristics.*

| Variable | Whole sample (n= 65) | MTE  (n= 33) | Waitlist  (n= 32) |  | p |
| --- | --- | --- | --- | --- | --- |
|  | N (%) | n (%) | n (%) | χ2 |  |
| Presence of trauma in childhood or adolescence |  |  |  |  |  |
| Victim of physical violence | 24 (36.9) | 14 (42.2) | 10 (31.3) | .87 | .35 |
| Victim of sexual abuse |  |  |  | .47 | .79 |
| Victim of sexual abuse (“yes” answer) | 22 (33.8) | 12 (36.4) | 10 (31.3) |  |  |
| Victim of sexual abuse (“I am not sure” answer) | 5 (7.7) | 3 (9.1) | 2 (6.3) |  |  |
| Has at least one family member with a mental health disorder | 29 (44.6) | 18 (54.5) | 11 (37.9) | 2.67 | .10 |
| Currently in psychological treatment | 28 (43.1) | 15 (45.5) | 13 (46.4) | .15 | .69 |
| Currently in psychopharmacological treatment | 20 (30.8) | 10 (30.3) | 10 (31.3) | .00 | .93 |
| Prior psychological or psychopharmacological treatment | 52 (80.0) | 31 (93.9) | 21 (65.6) | 8.14 | .00* |
| Diagnoses^a^ |  |  |  |  |  |
| Depression diagnosis |  |  |  | .50 | .47 |
| Minor depression | 8 (12.3) | 5 (15.2) | 3 (9.4) |  |  |
| Major depression | 57 (87.7) | 28 (84.8) | 29 (90.6) |  |  |
| Dysthymia | 6 (9.2) | 4 (12.9) | 2 (6.7) | .66 | .41 |
| Current generalized anxiety disorder | 40 (61.5) | 18 (54.5) | 22 (68.8) | 1.38 | .23 |
| Low-risk incidence of suicide | 15 (23.1) | 8 (24.2) | 7 (21.9) | .051 | .82 |
|  | M (SD) | M (SD) | M (SD) | t |  |
| Scores on questionnaires |  |  |  |  |  |
| PHQ-9  Range | 13.01 (4.5)  6-25 | 11.66 (3.6) | 14.40 (4.9) | -2.54 | .01* |
| EPDS  Range | 14.64 (4.1)  6-25 | 13.21 (3.7) | 16.1 (4.0) | -3.02 | .00* |
| PBQ  Range | 26.50 (13.0)  7-64 | 25.75 (12.1) | 27.28 (14.1) | -.46 | .64 |
| MSPSS  Range | 32.67 (9.0)  13-48 | 31.18 (7.0) | 34.2 (10.6) | -1.34 | .18 |
| EEP  Range | 5.99 (1.5)  3-9.60 | 6.24 (1.3) | 5.7 (1.7) | 1.29 | .19 |
| CEQ-6  Range | 42.93 (8.1)  10-50 | 41.96 (9.4) | 43.93 (6.5) | -9.76 | .33 |

Note. Abbreviations (alphabetical): χ2 = Statistic of χ2-test; M = Mean; n = Number of participants; p = p-value; SD = Standard deviation; t = Statistic of independent t-test.

* p < .05; ^a^ based on the MINI-international neuropsychiatric interview.

**Table S3**

*TWente Engagement with Ehealth Technologies Scale (TWEETS) descriptive analysis (n = 22; Intervention group only)*

| Item | Mean | SD | Score frequency (%) | | | | |
| --- | --- | --- | --- | --- | --- | --- | --- |
|  |  |  | 0 | 1 | 2 | 3 | 4 |
| MTE was part of my daily routine | 2.00 | .92 | 0 | 36.4 | 31.8 | 27.3 | 4.5 |
| MTE takes me little effort to use | 3.00 | 1.15 | 4.5 | 9.1 | 9.1 | 36.4 | 40.9 |
| I was able to use MTE as often as needed to improve my mood | 2.77 | .81 | 0 | 9.1 | 18.2 | 59.1 | 13.6 |
| MTE made it easier for me to work on improving my mood | 3.09 | .68 | 0 | 0 | 18.2 | 54.5 | 27.3 |
| MTE motivated me to improve my mood | 3.27 | .55 | 0 | 0 | 4.5 | 63.6 | 31.8 |
| MTE helped me to get more insight into my thoughts and emotions | 3.68 | .47 | 0 | 0 | 0 | 31.8 | 68.2 |
| I enjoyed using MTE | 3.14 | .77 | 0 | 0 | 22.7 | 40.9 | 36.4 |
| I enjoyed seeing the progress I made in MTE | 3.36 | .79 | 0 | 0 | 18.2 | 27.3 | 54.5 |
| MTE fits me as a person | 3.23 | .86 | 0 | 4.5 | 13.6 | 36.4 | 45.5 |
| Total (Mean) | 3.05 | .58 |  |  |  |  |  |

*Note.* TWEETS item and total scores range from 0 to 4, with higher values indicating higher engagement. Abbreviations (alphabetical): MTE = “Mamá, te entiendo” internet-based intervention; SD = Standard Deviation.

**Table S4**

*Client Satisfaction Questionnaire (CSQ-8) descriptive analysis (n = 22; Intervention group only)*

| Item | Mean | SD | Score frequency (%) | | | |
| --- | --- | --- | --- | --- | --- | --- |
|  |  |  | 1 | 2 | 3 | 4 |
| Quality of service (item 1) | 3.36 | .65 | 0 | 9.1 | 45.5 | 45.5 |
| Type of service (item 2) | 3.18 | .73 | 0 | 18.2 | 45.5 | 36.4 |
| Solves problems (item 3) | 2.68 | .64 | 0 | 40.9 | 50.0 | 9.1 |
| Recommend (item 4) | 3.59 | .59 | 0 | 4.5 | 31.8 | 63.6 |
| Amount of help (item 5) | 3.50 | .67 | 0 | 9.1 | 31.8 | 59.1 |
| Deal with problems (item 6) | 3.32 | .56 | 0 | 4.5 | 59.1 | 36.4 |
| Overall satisfaction (item 7) | 3.59 | .66 | 0 | 9.1 | 22.7 | 68.2 |
| Come back (item 8) | 3.45 | .73 | 0 | 13.2 | 27.3 | 59.1 |
| Total (Sum) | 26.68 | 4.3 |  |  |  |  |

*Note.* CEQ-8 total scores range from 8 to 32, with higher values indicating higher satisfaction; CEQ-8 items scores range from 1 to 4. Abbreviations: SD = Standard Deviation.

**Table S5**

*Key facilitators and barriers to the use of “Mamá, te entiendo” identified in the acceptability interviews.*

|  | Facilitators | Barriers |
| --- | --- | --- |
| Perception of Intervention | - Perceived as “support” and “accompanying.” - Approachable and surprisingly so due to the format. - Seen as a “moment of self-care”. |  |
| Content | - Comprehensive, down-to-earth, and well-organized. - Clear language and useful techniques. | - Some found certain technical language challenging, especially cognitive distortions. - Text length and complexity of content are perceived as challenging, particularly when tired or because of “baby brain”. - Limited exploration of other app sections than the modules. |
| Fictional mothers | - Real, representative, illustrative, and contributing to the normalization of experiences. |  |
| Homework exercises | - Valued for usefulness and aiding in internalizing content. |  |
| E-coach’s feedback | - Valued for being approachable, empathetic, and helpful. |  |
| Design and aesthetics | - Described as pretty, visually pleasing, and user-friendly. | - Not having progress tracking within the modules made it difficult to know where they left off. |
| Duration of intervention | - Adequate for completing the modules for most. | - Limited exploration of other app sections due to time constraints. |
| Objective achievement | - Contributed positively to mental health, reflective understanding, emotion regulation, and modification of dysfunctional thoughts. |  |

**Table S6**

*MINI depression diagnoses at baseline, post-intervention, and 1-month follow-up by group*

|  | MTE | | |  | WL | | |
| --- | --- | --- | --- | --- | --- | --- | --- |
| Depression diagnosis | T0  (n=33) | T1  (n=22) | T2  (n=22) |  | T0  (n=32) | T1  (n=30) | T2  (n=30) |
| No depression n, (%) | 0 | 10 (45.5%) | 15 (68.1%) |  | 0 | 13 (43.3%) | 19 (63.3%) |
| Minor depression, n, (%) | 5 (15.2%) | 5 (22.7%) | 2 (9.1%) |  | 3 (9.4%) | 8 (26.7%) | 1 (3.3%) |
| Major depression, n, (%) | 28 (84.8%) | 7 (43.8%) | 5 (22.7%) |  | 29 (90.6%) | 9 (30.0%) | 10 (33.3%) |

*Note.* Abbreviations (alphabetical): MTE = “*Mamá, te entiendo*” internet-based intervention; T0 = baseline; T1 = post-intervention; T2 = follow-up; WL = waitlist.

**Table S7**

*Paired t-test and Cohen’s d for changes from baseline at post-intervention and follow-up.*

|  |  |  |  | Post-intervention (T1) | | | | | |  | Follow-up (T2) | | | | | |
| --- | --- | --- | --- | --- | --- | --- | --- | --- | --- | --- | --- | --- | --- | --- | --- | --- |
| Variable | Analysis | Group |  | Cohen’s *d* (%95CI) | |  | t (*df*) |  | p | | Cohen’s *d* (%95CI) | |  | t (*df*) |  | p |
| PHQ9 | ITT | MTE |  | -0.31 (-0.68, 0.07) |  |  | -1.60 (23.14) |  | .12 |  | -0.25 (-0.59, 0.09) |  |  | -1.22 (21.09) |  | .23 |
|  |  | WL |  | -0.53 (-0.92, -0.11) | * |  | -2.92 (26.6) |  | .00 |  | -0.43 (-0.81, -0.01) | * |  | -2.43 (27.77) |  | .02 |
|  | CCA | MTE |  | -0.33 (-0.81, 0.14) |  |  | -1.59 (21) |  | .12 |  | -0.30 (-0.73, 0.14) |  |  | -1.44 (21) |  | .16 |
|  |  | WL |  | -0.53 (-0.93, -0.10) | * |  | -2.99 (29) |  | .00 |  | -0.40 (-0.79, -0.01) | * |  | -2.27 (29) |  | .03 |
| EPDS | ITT | MTE |  | -0.44 (-0.77, -0.11) | * |  | -2.17 (20.81) |  | .04 |  | -0.57 (-0.94, -0.21) | * |  | -2.91 (22.72) |  | .00 |
|  |  | WL |  | -0.63 (-0.96, -0.29) | * |  | -3.53 (27.67) |  | .00 |  | -0.74 (-1.00, -0.42) | * | | -4.13 (27.28) |  | .00 |
|  | CCA | MTE |  | -0.47 (-0.88, -0.07) | * |  | -2.28 (21) |  | .03 |  | -0.61 (-1.00, -0.21) | * |  | -2.96 (21) |  | .00 |
|  |  | WL |  | -0.58 (-0.92, -0.24) | * |  | -3.27 (29) |  | .00 |  | -0.72 (-0.99, -0.38) | * | | -4.03 (29) |  | .00 |
| EEP | ITT | MTE |  |  |  |  |  |  |  |  | 0.40 (0.06, 0.74) | * |  | 2.14 (24.59) |  | .04 |
|  |  | WL |  |  |  |  |  |  |  |  | 0.47 (0.10, 0.83) | * |  | 2.68 (28.88) |  | .01 |
|  | CCA | MTE |  |  |  |  |  |  |  |  | 0.50 (0.06, 0.93) | * |  | 2.42 (21) |  | .02 |
|  |  | WL |  |  |  |  |  |  |  |  | 0.41 (0.02, 0.78) | * |  | 2.28 (29) |  | .03 |
| PBQ | ITT | MTE |  |  |  |  |  |  |  |  | -0.51 (-0.84, -0.17) | * |  | -2.70 (24.42) |  | .01 |
|  |  | WL |  |  |  |  |  |  |  |  | -0.49 (-0.81, -0.04) | * |  | -2.82 (28.53) |  | .00 |
|  | CCA | MTE |  |  |  |  |  |  |  |  | -0.69 (-1.00, -0.25) | * |  | -3.34 (21) |  | .00 |
|  |  | WL |  |  |  |  |  |  |  |  | -0.54 (-0.84, -0.05) | * |  | -3.011 (29) |  | .00 |
| MSPSS | ITT | MTE |  |  |  |  |  |  |  |  | 0.64 (0.26, 0.99) | * |  | 3.126 (20.23) |  | .005 |
|  |  | WL |  |  |  |  |  |  |  |  | 0.34 (0.02, 0.64) |  |  | 1.942 (28.44) |  | .062 |
|  | CCA | MTE |  |  |  |  |  |  |  |  | 0.65 (0.20, 1.00) | * |  | 3.156 (21) |  | .005 |
|  |  | WL |  |  |  |  |  |  |  |  | 0.34 (-0.01, 0.68) |  |  | 1.888 (29) |  | .069 |

*Note.* The Cohen’s d CI was calculated via Bias-corrected and accelerated (BCA) bootstrapping.

Abbreviations (alphabetical): CCA = Complete case analyses; df = degrees of freedom; IIT = Intended-to-treat analyses; MTE = “*Mamá, te entiendo*” internet-based intervention; p = p-value; t = Statistic of paired t-test; WL = waitlist.

* p < .05

CONSORT 2010 checklist of information to include when reporting a pilot or feasibility trial

| Section/Topic | Item No | Checklist item | Reported |
| --- | --- | --- | --- |
| Title and abstract | | | |
|  | 1a | Identification as a pilot or feasibility randomised trial in the title | ✓ |
|  | 1b | Structured summary of pilot trial design, methods, results, and conclusions (for specific guidance see CONSORT abstract extension for pilot trials) | ✓ |
| Introduction | | | |
| Background and objectives | 2a | Scientific background and explanation of rationale for future definitive trial, and reasons for randomised pilot trial | ✓ |
|  | 2b | Specific objectives or research questions for pilot trial | ✓ |
| Methods | | | |
| Trial design | 3a | Description of pilot trial design (such as parallel, factorial) including allocation ratio | ✓ |
|  | 3b | Important changes to methods after pilot trial commencement (such as eligibility criteria), with reasons | N/A |
| Participants | 4a | Eligibility criteria for participants | ✓ |
|  | 4b | Settings and locations where the data were collected | ✓ |
|  | 4c | How participants were identified and consented | ✓ |
| Interventions | 5 | The interventions for each group with sufficient details to allow replication, including how and when they were actually administered | ✓* |
| Outcomes | 6a | Completely defined prespecified assessments or measurements to address each pilot trial objective specified in 2b, including how and when they were assessed | ✓* |
|  | 6b | Any changes to pilot trial assessments or measurements after the pilot trial commenced, with reasons | N/A |
|  | 6c | If applicable, prespecified criteria used to judge whether, or how, to proceed with future definitive trial | N/I |
| Sample size | 7a | Rationale for numbers in the pilot trial | ✓* |
|  | 7b | When applicable, explanation of any interim analyses and stopping guidelines | N/A |
| Randomisation: |  |  |  |
| Sequence  generation | 8a | Method used to generate the random allocation sequence | ✓ |
|  | 8b | Type of randomisation(s); details of any restriction (such as blocking and block size) | ✓ |
| Allocation  concealment  mechanism | 9 | Mechanism used to implement the random allocation sequence (such as sequentially numbered containers), describing any steps taken to conceal the sequence until interventions were assigned | ✓ |
| Implementation | 10 | Who generated the random allocation sequence, who enrolled participants, and who assigned participants to interventions | ✓* |
| Blinding | 11a | If done, who was blinded after assignment to interventions (for example, participants, care providers, those assessing outcomes) and how | ✓ |
|  | 11b | If relevant, description of the similarity of interventions | N/A |
| Statistical methods | 12 | Methods used to address each pilot trial objective whether qualitative or quantitative | ✓ |
| Results | | | |
| Participant flow (a diagram is strongly recommended) | 13a | For each group, the numbers of participants who were approached and/or assessed for eligibility, randomly assigned, received intended treatment, and were assessed for each objective | ✓ |
|  | 13b | For each group, losses and exclusions after randomisation, together with reasons | ✓ |
| Recruitment | 14a | Dates defining the periods of recruitment and follow-up | ✓ |
|  | 14b | Why the pilot trial ended or was stopped | N/A |
| Baseline data | 15 | A table showing baseline demographic and clinical characteristics for each group | ✓ |
| Numbers analysed | 16 | For each objective, number of participants (denominator) included in each analysis. If relevant, these numbers  should be by randomised group | ✓ |
| Outcomes and estimation | 17 | For each objective, results including expressions of uncertainty (such as 95% confidence interval) for any  estimates. If relevant, these results should be by randomised group | ✓ |
| Ancillary analyses | 18 | Results of any other analyses performed that could be used to inform the future definitive trial | ✓ |
| Harms | 19 | All important harms or unintended effects in each group (for specific guidance see CONSORT for harms) | N/I |
|  | 19a | If relevant, other important unintended consequences |  |
| Discussion | | | |
| Limitations | 20 | Pilot trial limitations, addressing sources of potential bias and remaining uncertainty about feasibility | ✓ |
| Generalisability | 21 | Generalisability (applicability) of pilot trial methods and findings to future definitive trial and other studies | ✓ |
| Interpretation | 22 | Interpretation consistent with pilot trial objectives and findings, balancing potential benefits and harms, and  considering other relevant evidence | ✓ |
|  | 22a | Implications for progression from pilot to future definitive trial, including any proposed amendments | ✓ |
| Other information | | |  |
| Registration | 23 | Registration number for pilot trial and name of trial registry | ✓ |
| Protocol | 24 | Where the pilot trial protocol can be accessed, if available | ✓ |
| Funding | 25 | Sources of funding and other support (such as supply of drugs), role of funders | ✓ |
|  | 26 | Ethical approval or approval by research review committee, confirmed with reference number | ✓ |

N/A: not applicable; N/I: not included in the published study protocol.

*Items marked with an asterisk (*) are presented in a summarized form in this article due to word count constraints. A more detailed description of these elements can be found in the protocol paper, which is cited in this article: Franco, P., Olhaberry, M., Cuijpers, P., Kelders, S., & Muzard, A. (2023). App-based intervention for reducing depressive symptoms in postpartum women: Protocol for a feasibility randomized controlled trial. *Internet Interventions, 32*, 100616. <https://doi.org/10.1016/j.invent.2023.100616>.

Eldridge SM, Chan CL, Campbell MJ, Bond CM, Hopewell S, Thabane L, et al. CONSORT 2010 statement: extension to randomised pilot and feasibility trials. BMJ. 2016;355. This is an Open Access article distributed in accordance with the terms of the Creative Commons Attribution (CC BY 3.0) license (<http://creativecommons.org/licenses/by/3.0/>), which permits others to distribute, remix, adapt and build upon this work, for commercial use, provided the original work is properly cited.
